# A Retrospective Multicentric Study of Electrochemotherapy in the Treatment of Feline Nasal Planum Squamous Cell Carcinoma

**DOI:** 10.3390/vetsci8030053

**Published:** 2021-03-22

**Authors:** Petra Simčič, Alessio Pierini, George Lubas, Ron Lowe, Valentina Granziera, Raimondo Tornago, Fabio Valentini, Giulia Alterio, Matteo Cochi, Marcelo Monte Mor Rangel, Krishna Duro de Oliveira, Jennifer Ostrand Freytag, Priscila Gil Quadros, Enrico Sponza, Francesca Gattino, Joseph A. Impellizeri, Filippo Torrigiani

**Affiliations:** 1 Department of Veterinary Sciences, University of Pisa, Via Livornese Lato Monte, San Piero a Grado, 56122 Pisa, Italy; pierini.alessio2004@gmail.com (A.P.); george.lubas@unipi.it (G.L.); 2 PetCancerVet, 61 Wetherby Road, Knaresborough, North Yorkshire HG5 8LH, UK; expetcancervet@gmail.com (R.L.); valentinagranziera89@gmail.com (V.G.); 3 Meranese Veterinary Centre, Via J. Speckbacher, 15/B, 39012 Merano, Italy; raimondotornago@gmail.com; 4 Via Marco Cornelio Cetego 20, 00177 Rome, Italy; f.valentini@oncovet.it (F.V.); g.alterio@oncovet.it (G.A.); m.cochi@oncovet.it (M.C.); 5 Vet Câncer—Animal Oncology and Pathology, AL Jauaperi, 732 Moema, São Paulo, SP 04523-013, Brazil; vetcancerbrasil@gmail.com (M.M.M.R.); krishnaoliveira@yahoo.com.br (K.D.d.O.); contato@vetcancer.com.br (J.O.F.); priscilaq54@gmail.com (P.G.Q.); 6 Veterinary Facility Dr. Enrico Sponza, Via Pra’ 39/r, 16157 Genova, Italy; sponzaenrico@gmail.com (E.S.); francesca.gattino@unito.it (F.G.); 7 Veterinary Oncology Services, PLLC at Guardian Veterinary Specialists, Brewster, NY 10509, USA; oncologyvet@yahoo.com; 8 Department of Comparative Biomedicine and Food Science, University of Padova, Viale dell’Università 16, 35020 Legnaro, Italy; filippo.torrigiani@unipd.it

**Keywords:** feline, squamous cell carcinoma, nasal planum, electrochemotherapy, bleomycin, toxicity, retrospective, multicentric

## Abstract

Feline squamous cell carcinoma (SCC) is currently treated with surgery, radiation therapy and electrochemotherapy (ECT). Both the efficacy and/or safety of ECT were evaluated as a sole therapy with bleomycin to treat feline nasal planum SCC (npSCC). Sixty-one cats were enrolled. Local treatment response was evaluated as complete remission (CR), partial remission (PR) or stable disease (SD). Recurrence rate (RR), disease-free interval (DFI) and progression free survival (PFS) were calculated. A six-point scale was used for ECT toxicity. The median tumor size was 1.5 cm. CR was achieved in 65.6% of cases, PR in 31.1% and SD in 3.3%. The overall response rate was 96.7%, RR was 22.5%, median DFI was 136 days, and median PFS was 65.5 days. ECT toxicity was ≤2 in 51% of cats. Tumor recurrence/progression (*p* = 0.014) and local treatment response (PR: *p* < 0.001; SD: *p* < 0.001) influenced survival time. Cats with toxicity >2 showed a higher probability of tumor recurrence/progression. Tumor-related death was higher in cats with PR (*p* < 0.001) and recurrence/progression (*p* = 0.002), in ECT treatment with 1 Hz (*p* = 0.035) and 1200 V/cm (*p* = 0.011) or 1300 V/cm (*p* = 0.016). Tumor size influenced local treatment response (*p* = 0.008) and toxicity (*p* < 0.001). ECT is an effective treatment for feline npSCCs and should be considered as the first-line procedure for low-stage tumors.

## 1. Introduction

Squamous cell carcinoma (SCC) is a malignant tumor arising from epidermal cells with differentiation to keratinocytes (squamous cells). In the skin, squamous cells can be found in the epidermis, the dermis, as well as nail beds and foot pads, along with the oral cavity and the esophagus. SCCs are the most common tumors in cats and account for approximately 10.4% of feline skin tumors above all involving the nasal planum, with ear pinnae and eyelids also affected [1,2,3]. Cats older than 10–12 years are usually at higher risk [2,4].

In cats, cutaneous SCCs are mainly associated with chronic solar exposure (UV light)—more precisely, ultraviolet B radiation. Lightly pigmented, white or colored cats with white areas are at greater risk, while long-haired cats have a reduced probability of developing a SCC. The lesions usually arise in alopecic areas, such as the ears, eyelids, nasal planum and temporal areas [2]. Chronic solar exposure is usually involved in the progression from actinic changes to in-situ SCC and then invasive SCC [4]. Histologically, the invasion of the basement membrane by neoplastic squamous cells is the main feature that discriminates overt SCC from preneoplastic changes (i.e., actinic changes). Neoplastic cells show a wide range of malignant histological features, such as cellular pleomorphism, karyomegaly and mitotic figures, which are usually more prominent in poorly differentiated neoplasms, whilst in well-differentiated SCCs, “keratin pearls” are commonly formed [5]. In more than 50% of cats with SCC, mutations on the TP53 gene and overexpression of the P53 protein were found [6,7].

Clinically, SCCs can appear as plaque-like to papillary, crateriform to fungiform lesions which may be ulcerated and erythematous with crusts [4,6]. Clinical staging for cats with SCCs follows the WHO tumor–node–metastasis staging system (TNM) [6]. Feline SCCs are usually locally aggressive but slow to metastasize [4]. If they metastasize, the regional lymph nodes and the lungs are most likely affected [2].

Treatment success depends on clinical stage, tumor invasiveness, location and the extent of the lesions. The treatment is more effective in the initial onset of the disease, regardless of the approach. Radical surgical excision provides the best cure rates in the treatment of nasal planum SCC (npSCC); however, clean surgical margins are crucial to achieve local tumor control. This goal is not always achievable, especially for higher-stage lesions (namely T3 and T4) [2,4,8,9]. Moreover, cosmetic, short- and long-term functional defects are of great concern when attempting an aggressive extirpation with a wide-margin nosectomy. For these reasons, numerous local antitumor techniques have been applied in the treatment of npSCC, which include cryosurgery, radiation therapy, photodynamic therapy, intralesional chemotherapy and electrochemotherapy. Systemic chemotherapy has also been used; however, it is usually limited to advanced-stage cases and results have been underwhelming [2,4,10,11,12,13,14,15,16,17,18,19,20,21].

This paper investigates electrochemotherapy (ECT), which was first described in 1991 and has shown encouraging results in veterinary oncology [22,23]. This treatment combines the use of reversible electroporation (short high-voltage electric pulses) with cytotoxic drugs [9,24]. The two most studied and effective agents are bleomycin and cisplatin [25]. These two drugs are hydrophilic and do not easily penetrate the cell membrane; however, upon entering the cells, they are highly cytotoxic. The application of permeabilizing electric impulses leads to an increase in the uptake of the normally non-permeable or poorly permeable cytotoxic drugs [22,24].

Drug accumulation in targeted cells is due to increased membrane permeability, which finally induces cell death [26]. Two other mechanisms involved in cell death are vascular lock, which causes vasoconstriction and decreased blood flow into the tumor, and vascular disrupting mechanism, which provokes death of the vascular endothelial cells [26]. Some studies have shown that ECT has a stimulatory impact on the patient’s immune system due to the massive antigen shedding from the treated area. The immune response in some cases may also result in a systemic effect, known as an abscopal response, on distant, nontreated nodules [27,28].

ECT is mostly used for treating histologically different, locally invasive, superficial tumors, although it has also been used to treat visceral tumors, such as liver metastasis and colorectal cancer in human patients [22]. In small-animal clinical oncology, ECT has mainly been used to treat locally invasive superficial tumors such as mast cell tumors, soft tissue sarcomas, perianal neoplasms, oral squamous cell carcinomas, melanomas and nasal tumors in dogs. The feline literature is mostly restricted to cutaneous SCCs and soft-tissue sarcomas [29,30,31,32,33,34,35].

ECT is minimally invasive, with minor or no side effects. It can be repeated several times, requires general anesthesia and the costs are lower than other treatment approaches, such as radiation therapy [21]. ECT can also be used in combination with several other therapeutic approaches, such as surgery, radiation therapy and gene therapy [30,36,37]. ECT has been used as a single-treatment approach [9,38] or combined with the current standard of care approaches for the treatment of nasal planum SCC (npSCC), such as surgery [33,39]. One report also described a case in which it was combined with radiation therapy, with some local complications [40].

The aim of this multicentric and retrospective study was to evaluate the efficacy and safety of ECT in the treatment of feline npSCC in a case series.

## 2. Materials and Methods

### 2.1. Patient Selection

This multicentric study included 61 privately owned cats diagnosed with npSCC. The study population was retrospectively enrolled, during the period from August 2005 to May 2020, from six different veterinary facilities as follows:

“Vet Câncer”, Sao Paolo, Brasil (VCB), 26 patients (42.6%); “Veterinary Facility Dr. Enrico Sponza”, Genova, Italy (SVG), 12 patients (19.7%); “Ashleigh Vet Clinic”, Knaresborough, UK (AVC), 10 patients (16.4%); “Oncovet Group”, Rome, Italy (OVG), 10 patients (16.4%); “Meranese Veterinary Center”, Bolzano, Italy (CVM), 2 patients (3.3%); and “Veterinary Oncology Services, PLLC at Guardian Veterinary Specialists”, New York, USA (GVS), 1 patient (1.6%).

From the clinical records available at each facility, details on the study population (breed, age and sex) and information on tumor size (caliper measurements) were collected.

ECT was offered to the owners as an option alongside standard treatment approaches, such as surgery and/or radiation therapy. Informed consent illustrating the entire ECT procedure was signed by each owner. ECT as the sole therapy was required for inclusion in this study.

### 2.2. Staging

The patients were diagnosed with npSCC using cytology (17/61) or histology (34/61) and, in some cases, with both (10/61). Patient staging was performed in cats that already had medical records with complete information regarding clinical examination, fine needle aspirates of the regional lymph nodes if enlarged and three view thoracic radiographs. Skull radiographs were performed depending on the clinician in charge at the time of the diagnostic work-up.

Only cats without metastatic disease in the regional lymph nodes or lungs were included in the study.

Since ECT was performed under general anesthesia, a complete blood count, coagulation profile and biochemistry profile were performed to rule out underlying diseases at the clinician’s discretion. All the cats, except two showing unrelated chronic kidney disease (CKD, IRIS II), had unremarkable blood chemistry parameters.

### 2.3. Electroporators, Electrical Parameters and Anesthesia Protocol

The equipment and their electrical characteristics (frequency and amplitude to electrode distance ratio) used for each case treated are listed in Table 1. Although different pulse generators were used in this multicentric study, all delivered 8 monophasic square pulses of 100 µs each. The different protocols used for general anesthesia depended on the veterinary facility and the clinician in charge (see Table 1).

### 2.4. Treatment (ECT) Protocol

ECT was combined with the standard dose of bleomycin (15,000 UI/m^2^, European Farmacopeia) except for one cat, treated at GVS, for which a lower dosage was used (10,000 UI/m^2^). Based on recent publications, lower doses of bleomycin (10,000 IU/m^2^ rather than 15,000 UI/m^2^) were shown to be equally effective in clinical response. One researcher (JAI) used the lower dosing regimen as a standard approach with ECT [41,42]. In all cases, bleomycin was administered intravenously. Eight minutes after drug administration, the clinician proceeded with the application of electric pulses.

All the cats were treated with ECT alone. A single ECT treatment was performed in 39/61 (63.9%) cats. The remaining cats, 22/61 (36.1%), had more than one ECT due to recurrence, presence of stable disease (SD) or to potentiate treatment efficacy (Table 2).

After ECT, cats were administered additional support therapy if needed. The post-treatment plan differed at each facility (Table 2).

### 2.5. Follow-Up and Treatment Outcome

ECT patients were monitored once or twice a week for the first four weeks, followed by a monthly checkup for an additional three months and then every 3–4 months. The follow-up visit was performed by the referral veterinarian. Some clinicians also photographed the healing process and the progression of the patients.

The local response to the treatment was evaluated as: (CR) complete remission and no sign of the primary tumor; (PR) partial remission, i.e., at least 30% reduction in the tumor but not the disappearance of the mass, and (SD) stable disease where the size of the tumor showed no reduction or enlargement (less than 30% reduction in size (PR) or less than 20% increase in size (PD)—progressive disease) [43].

Treatment response rate, survival time (time from first treatment to the day of the last checkup or death) and median survival time (MST) were calculated. In cats that experienced CR and subsequent local recurrence, the recurrence rate (RR) and disease-free interval (DFI) were calculated. DFI was defined as the time from first treatment to the day of local recurrence or evidence of metastasis. In cats that experienced PR or SD, progression free survival (PFS) was defined as the time from first treatment to the day of local progression of the disease or evidence of metastasis. Finally, the treatment outcome of the patients was evaluated: alive without tumor, alive with tumor, dead without tumor and dead with tumor. MST was calculated for the deceased cats and median follow-up was calculated for the cats that were still alive at the conclusion of the study period.

Local toxicity was retrospectively evaluated after the first ECT treatment, using a 6-point toxicity score, developed by Lowe et al., where 0—no toxicity, 1—mild swelling, 2—swelling/necrosis <1 cm, 3—severe swelling, 4—deep necrosis and 5—severe swelling and tissue loss [29].

### 2.6. Statistics

The data were initially analyzed as descriptive statistics. Size, age, DFI, PFS and survival times were analyzed as non-parametric, continuous variables and presented as median and range. Categorical variables (gender, breed and number of treatments) and continuous variables (pulse frequency, pulse to electric distance ratio, local treatment response and toxicity score) were presented as absolute and relative frequencies. Local treatment response was divided at the beginning into three categories as follows: CR, PR and SD. However, due to only a few cases with SD, PR and SD were considered as a single category when local treatment response to ECT was investigated. The toxicity score for each cat was subtyped into two groups: toxicity score ≤2 and >2. Alive cats with neither tumor progression/recurrence nor metastasis and cats that died due to tumor-unrelated causes were censored at the last follow-up. The only cat treated at GVS was not included in the following statistical analysis, since a specific ECT protocol was applied (Table 1).

The data were evaluated by inferential statistics. Kaplan–Meier curves and two-sided log-rank tests were used as the univariate analysis to compare survival times and times of recurrence/progression occurrence among categorical and continuous variables. Cox regression (proportional hazard regression) analysis was used for continuous variables (age and size), which, if significant, with a *p* < 0.05, were entered into multivariate analysis (Cox regression, proportional hazard regression).

A univariate analysis (binary logistic regression analysis) was used to establish the relationship between gender, pulse frequency, amplitude to electrode distance ratio, presence of recurrence/progression, age and tumor size and the tumor specific survival, local treatment response and the toxicity score. Variables with a *p* < 0.05 on univariate analysis were entered into multivariate analysis (binary logistic regression analysis).

For each continuous variable significantly associated with survival, local treatment response and toxicity, a receiver operating characteristic (ROC) analysis was performed and the area under the curve (AUC) was calculated. Diagnostic cut-offs and their sensitivity and specificity were determined according to the maximum Youden index.

All statistics were analyzed with IBM SPSS^®^ Statistics.

## 3. Results

The study population of 61 cats was mostly represented by the domestic short hair (DSH) (56/61, 91.8%) and only five cats (8.2%) were domestic long hair (DLH). The median age of the study population was 11 years old and ranged from 5 to 17 years. All cats included had been neutered, and they were almost equally distributed between females (31/61, 50.8%) and males (30/61, 49.2%). The median tumor size was 1.5 cm (range 0.2–6.0 cm).

Forty out of 61 cats (65.6%) experienced CR and 19/61 cats (31.1%) experienced PR. The overall response rate to the treatment for the study population was 96.72% (59/61). Only two cats had SD (2/61, 3.3%).

The survival time of the whole study population ranged from 13 to 2929 days, with an MST of 286 days. Overall, the RR for cats with CR (40/61, 65.6%) was 22.5% (9/40). Cats with local tumor recurrence after CR had a median DFI of 136 days (range 29–302 days). Ten out of 61 cats (16.4%) that achieved PR (9/10) and SD (1/10) had local progression with a median PFS of 65.5 days (range 16–264 days).

At the end of the study period, 14/61 (23%) cats were still alive and 47/61 (77%) cats died. Among the cats that were still alive at the end of the study period, twelve (12/14, 19.7%) cats were in CR and 2/14 (3.3%) still had a tumor after treatment. In the group of cats that died during the study period (47/61.8%), twenty-four (24/47, 39.3%) died without a tumor with CR, and twenty-three (23/47, 37.7%) died with a tumor with PR or SD. The MST for cats that died without tumor was 872 days (22-2,929 days) and 193 days (13–362 days) for those that died with a tumor. The median follow-up for cats still alive at the end of the study period was 394 days (range 29–2798 days).

The local treatment toxicity score was ≤2 in 31/61 (51.0%) patients. Of these, 14/61 (23.0%) cats did not show any toxicity (grade 0), 2/61 cats (3.3%) showed toxicity grade 1, and 15/61 cats (24.6%) showed toxicity grade 2. The rest of the cats (30/61, 49.0%) showed a toxicity score >2, as follows: 4/61 (6.6%) grade 3, 16/61 (23.0%) grade 4 and 12/61 (19.7%) grade 5 (Table 2).

**Figure 1:**
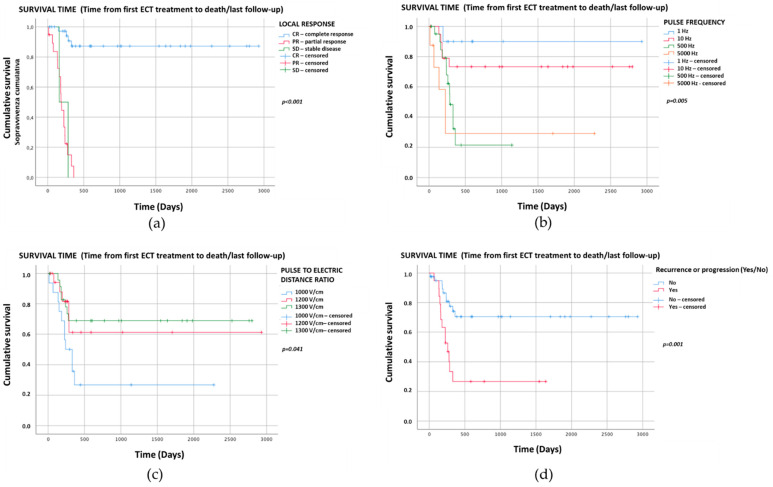
Kaplan–Meier graphs of the four categorical variables with *p* < 0.05 showing survival times (days). The comparison of survival curves with the log-rank test for (**a**) local treatment response was *p* < 0.001 (CR: median NR, 95%CI NR; PR: median 193, 95%CI 168–218; SD: median 160, 95%CI NR); (**b**) pulse frequency *p* = 0.005 (1 Hz: median NR, 95%CI NR; 10 Hz: median NR, 95%CI NR; 500 Hz: median 286, 95%CI 238–334; 5000 Hz: median 225, 95%CI 124–326); (**c**) amplitude to electrode distance ratio *p* = 0.041 (1000 V/cm: median 240, 95%CI 115–365; 1200 V/cm: median NR, 95%CI NR; 1300 V/cm: median NR, 95%CI NR); (**d**) recurrence/progression *p* = 0.001 (recurrence/progression: median 260, 95%CI 165–355; no recurrence/progression: median NR, 95%CI NR). NR—Not reached.

The Kaplan–Meier graphs for survival time (days) are reported in Figure 1a–d. Only the significant statistical comparisons of Kaplan–Meier survival curves with the log-rank analysis are presented: local treatment response (*p* < 0.001) (Figure 1a), pulse frequency (*p* = 0.005) (Figure 1b), amplitude to electrode distance ratio (*p* = 0.041) (Figure 1c) and recurrence/progression (*p* = 0.001) (Figure 1d).

The Cox regression analysis for continuous variables showed significant results only for tumor size (*p* = 0.005, OR 1.48, 95%CI 1.12–1.94).

The multivariate analysis for survival time was significant only for recurrence/progression (*p* = 0.014, OR 3.18, 95%CI 1.26–8.02) and local treatment response (CR as term of comparison: PR, *p* < 0.001, OR 23.97, 95%CI 7.3–78.8; SD, *p* < 0.001, OR 29.42, 95%CI 4.72–183.15).

**Figure 2:**
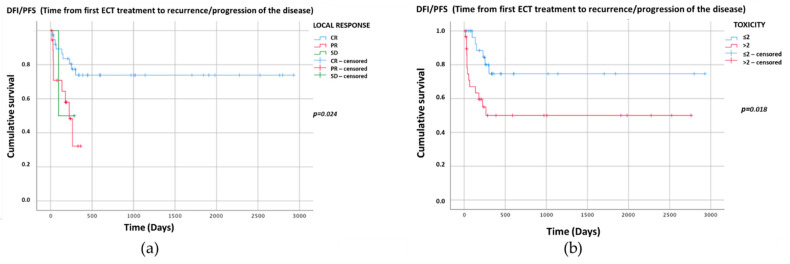
Kaplan–Meier graphs of the two categorical variables with *p* < 0.05 showing DFI/PFS (days). The comparison of survival curves with the log-rank test for (**a**) local treatment response *p* = 0.024 (CR: median NR, 95%CI NR; PR: median 225, 95%CI 139–311; SD: median 97, 95%CI NR); and (**b**) toxicity p = 0.018 (≤2: median NR, 95%CI NR, >2: median NR, 95%CI NR). NR—Not reached.

The Kaplan–Meier graphs for recurrence/progression (DFI/PFS) (days) are reported in Figure 2a,b. Only the significant statistical comparisons of Kaplan–Meier survival curves with the log-rank analysis are presented: local treatment response (*p* = 0.024) (Figure 2a) and toxicity score (*p* = 0.018) (Figure 2b).

The Cox regression analysis for continuous variables showed significant results only for tumor size (*p* = 0.004, OR 1.47, 95%CI 1.13–1.92). The multivariate analysis for DFI/PFS was significant only for toxicity score (toxicity ≤2 as reference category: *p* = 0.025; toxicity >2 OR 3.03, 95%CI 1.15–7.99).

Data from the univariate analysis and multivariate analysis used to establish the relationship between variables are presented in Table 3 for tumor-specific survival, Table 4 for local treatment response and Table 5 for toxicity. Local treatment response and recurrence/progression were independently associated with tumor-specific survival. Tumor size was associated with local treatment response. Tumor size >1.7 cm was associated with higher risk of PR/SD (AUC 0.79, 95%CI 0.67–0.91, *p* < 0.001, sensitivity 76%, specificity 72%). Tumor size >1.1 cm was associated with higher risk of toxicity score >2 (AUC 0.88, 95%CI 0.78–0.98, *p* < 0.001, sensitivity 93%, specificity 83%).

## 4. Discussion

The present retrospective study evaluated the efficacy and safety of ECT in the treatment of feline npSCC in a group of 61 cats. To date, only a few studies have been published on this topic, with a limited study population [9,32,38,44].

As with all facial SCCs, npSCC can also be locally invasive, with the outcome of the treatment approaches influenced by the stage and invasiveness of the tumor, with an improved outcome in lower-stage lesions (such as Tis and T1) [9]. Because of the major cosmetic and functional defects after surgery, nosectomy, which was considered the standard care for the treatment of canine npSCCs, should not be considered as the first-choice treatment for these tumors in cats when ECT is available [2]. Studies describing the use of cryosurgery have reported CR rates of up to 84% with fairly long response durations [10]. Other reports, however, have reported high recurrence rates (up to 73%) even for low-stage tumors, due to the impossibility of the evaluation of surgical margins after cryosurgery [4,10,11]. Another major limitation of cryosurgery for feline npSCCs is the multiple treatments needed in most cases [4,10,11].

Radiation therapy has been used to treat npSCCs in cats using many different protocols, including orthovoltage, megavoltage and proton beam irradiation. The main limitations of radiation therapy include the multiple anesthesias needed for treatment planning and delivery, the occurrence of early and late side effects after treatment, and the high cost [2,4,11,12,15,21].

Brachytherapy with strontium-90 has shown very good results, with favorable CR rates in two studies [13,14]. However, when the treatment was evaluated in a larger population, the occurrence of new lesions outside the radiation field penumbra was reported in 33% of patients [14]. In a recent study, the response to treatment was 100%, with a median DFI of 916 days [21].

Photodynamic therapy has also been used in feline npSCCs with excellent results. Although it can be safely repeated, when applied to deep seated tumors, the recurrence rate is high [16,17]. With this treatment, the photosensitizer can be administered both topically and systemically. When administered topically in feline SCCs, 85% of cats in the study obtained CR; however, 51% of them had tumor recurrence after a median time of 157 days [16]. Conversely, when the photosensitizer was administered intravenously, the response rate was 100%, with a 20% recurrence rate. One year after the treatment, 75% of the population maintained local control [17].

ECT, the topic of the present paper, is a novel treatment that combines both anti-tumor and favorable cosmetic effects at a reasonable cost, and it is increasingly used in veterinary oncology [9,22].

In a report by Spugnini et al. including nine cats with mostly npSCC treated with ECT combined with intratumoral administration of bleomycin, complete local control was obtained in 77.7% cats, which lasted for three years. Only two cats in the study died with local tumor recurrence [38].

Tozon et al. described a single ECT session coupled with systemic bleomycin for the treatment of SCCs located on the head in a population of 11 cats. The authors reported that CR was achieved in 81.8% of the population, with only two cats experiencing tumor recurrence, two and eight months, respectively, after achieving CR [9].

In a second study, Spugnini et al. reported the use of ECT coupled with systemic bleomycin for the treatment of feline periocular SCC and advanced SCC of the head. In this report, an overall response rate of 89% (21/47 CR and 2/47 PR) with a median PFS of 30.5 months was achieved [32].

Dos Anjos et al. compared two ECT protocols, using the standard dose of bleomycin (15,000 UI/m^2^) and a reduced dose (10,000 UI/m^2^) in cats with cutaneous SCC, treated with ECT alone. A group of 56 cats was included in the study, divided into two groups: 22 treated with the standard dose and 34 with the lower dose of bleomycin. The response rate and median DFI were 87.4%, 240 days and 100%, 210 days, respectively [44].

To the best of our knowledge, ours is the first study on the use of ECT alone on a large population of cats affected by SCC located exclusively on the nasal planum.

We combined ECT with the systemic administration of bleomycin, achieving a very good response rate with low RR or progression of the disease. The population consisted of 61 cats with non-metastatic npSCC, with a median tumor size of 1.5 cm. Similar data on tumor size have been reported in other studies of ECT in feline SCCs [9,32,38].

ECT was used as the sole therapy in all 61 cats, 96.7% of which responded to the treatment and 3.3% remained in SD. CR was achieved by 65.5% and PR by 31.1% of the cats included in the study. Our data on the treatment response rate are similar to, if not slightly better than, previous studies that describe ECT in feline SCCs, in which the RR ranged from 77 to 100%. The degree and completeness of the response also varies among studies and greatly depends on the clinical stage and tumor size [9,32,38,44]. Spugnini et al. (2015) obtained a CR rate of 44.6% but they included periocular and advanced SCCs of the head. However, when lower-stage tumors were included, CRs up to 81.8% were reported. Caution is needed when comparing our data with the previous literature, as this is the first study that only considers feline SCCs located on nasal planum treated with ECT. In our study, most of the patients (63.9%) required only one ECT session, which is in accordance with previously published studies [9,32,38,44]. However, 22 cats (36.1%) were treated more than once (Table 2). Multiple ECT sessions were mostly performed in cats that responded to the first ECT with PR or SD, and in cats that resulted in CR after one treatment but then had a recurrence. Among those cats treated with multiple ECT sessions, five patients received more than one ECT even if CR had been obtained after the first session, in order to potentiate the CR. This decision was taken by the clinicians in charge when a possible recurrence of the disease was suspected, based on the appearance of the tumor. In this study, the overall MST was 286 days (range 13–2929 days) and the overall RR for cats in CR was quite low (22.5%), with a median DFI of 136 days (range 29–302 days). Cats with local disease progression (10/61) had a median PFS of 65.5 days (range 16–264 days).

Local treatment toxicity was assessed using a previously published, subjective six-point scale [29]. This is the first study where this grading system has been applied in cats for the evaluation of local treatment toxicity due to ECT, as, until now, it has only been reported in canine tumors treated with ECT [29,30,31]. In the present study, local treatment toxicity after the treatment was absent or mild (score ≤ 2) in 51% of patients. However, the remaining 49% of our study population had a toxicity score of >2. Although an objective comparison with the literature is difficult due to the subjective nature of this toxicity scoring system, our data regarding local treatment toxicity are consistent with findings previously described in other studies on feline SCCs [9,32,38].

Kaplan–Meier curves showed that local treatment response (*p* < 0.001) (Figure 1a), pulse frequency (*p* = 0.005) (Figure 1b), amplitude to electrode distance ratio (*p* = 0.041) (Figure 1c), presence of recurrence or progression (*p* = 0.001) (Figure 1d) and tumor size (*p* = 0.005) significantly influenced the survival time.

From the multivariate analysis, the two variables with the highest influence on the survival time and with an increased probability of cats dying from tumor were the presence of tumor recurrence or progression of the disease (*p* = 0.014) and the local treatment response. Cats with PR (*p* < 0.001) or SD (*p* < 0.001) had a 23.97- and 29.42-times higher probability of dying of tumor-related causes, respectively, compared to the cats in CR. This is not surprising since cats with recurrence or progression of the disease tended to demonstrate a poorer prognosis in most of the published studies [44].

Recurrence or progression of the disease was significantly influenced by the local treatment response (*p* = 0.024) (Figure 2a), tumor size (*p* = 0.004) and toxicity score (*p* = 0.018) (Figure 2b). While a correlation between local treatment response and tumor size with recurrence or progression of the disease appears logical, and has been confirmed in the literature also considering other treatment approaches, the link between treatment outcome and local toxicity is surprising. In fact, the multivariate statistical analysis showed that cats with a toxicity score of >2 had a three-times higher probability of having a tumor recurrence or progression of the disease than cats with a toxicity score of ≤2 [9,32,44,45,46]. To the best of our knowledge, to date, no association between a higher number of local side effects and the recurrence or progression of the disease in tumors treated with ECT has been described. These data highlight the possibility that higher toxicity could influence the efficacy of the treatment and should be considered in future studies.

Death due to the presence of the tumor was significantly higher in cats with PR (*p* < 0.001) compared to the cats that achieved CR. Moreover, cats treated with 1 Hz (*p* = 0.035) had a lower chance (OR 0.07) of dying from a tumor compared to the cats treated with 5000 Hz. Similarly, cats treated with 1200 V/cm (*p* = 0.011) and 1300 V/cm (*p* = 0.016), compared to the cats treated with 1000 V/cm, had a higher probability of tumor-related death. Lastly, cats with recurrence or progression (*p* = 0.002) were logically more susceptible to tumor-related death. The impact of the local treatment response and of the recurrence or progression was also confirmed in the multivariate logistic binary regression (Table 3).

Local treatment response was influenced by pulse frequency (*p* = 0.035), meaning that cats treated with 1 Hz had a higher probability of achieving CR than cats treated with 5000 Hz. This is quite surprising as studies on both human and veterinary patients have proven that the application of 1-Hz or 5000-Hz pulses does not influence the clinical outcome. In light of our findings, data should be carefully considered and confirmed—ideally with a prospective, comparative study [26,47]. Cats with a toxicity score of ≤2 had a higher probability of achieving CR compared to cats with a toxicity score of >2. Local treatment response was also influenced by size (*p* = 0.001). The multivariate analysis confirmed that local treatment response was highly influenced by tumor size (*p* = 0.008) (Table 4).

Local treatment toxicity was influenced by pulse frequency for the frequency of 500 Hz leading to lower toxicity compared to 5000 Hz (*p* = 0.008). This finding has also never been reported in the literature and is possibly influenced by the multicentric nature of our study. In preclinical models, pulse frequency has been reported to influence the number and intensity of muscular contractions and therefore possible treatment-associated pain, which would be difficult to evaluate in veterinary patients [48,49]. In addition, the amplitude to electrode distance ratio also had an impact on the toxicity of the treatment, showing that tumors treated with 1300 V/cm showed higher toxicity than those treated with 1000 V/cm (*p* = 0.01). This result was also found by Torrigiani et al. (2019); however, it has not been reported elsewhere. Tumor size also significantly influenced the toxicity score (*p* < 0.001) (Table 5), which is in agreement with the literature on humans. In fact, one study describing the risk factors of pain associated with ECT treatment of cutaneous metastasis of various cancers found that the size of metastasis was associated with a higher pain score. The authors speculated that large, and possibly necrotic, metastases would also take more time to heal. In light of our results, the owners of cats with larger npSCCs should be warned of the risk of more severe side effects [48].

Lastly, as already reported in other tumor types, size was closely associated with treatment outcome. In our case, the cut-off value that guaranteed a higher probability of CR was <1.7 cm [31,50]. Since ECT causes apoptosis and tissue necrosis, the association between tumor size and a higher risk of a toxicity score of >2 when the tumor size is >1 cm does not seem surprising. The larger the tumor treated with ECT, the larger the area that undergoes tissue necrosis, and the higher the chances of local toxicity reaching scores above 2. However as reported above, more studies should be performed to confirm this hypothesis.

The main limitation of the present study is the retrospective nature of the collected data. In addition, the use of different types of electroporators as well as different electroporation protocols due to the multicentric nature of the study prevents definitive conclusions from being drawn on the ideal ECT protocol for cats with npSCC. A study on a larger population using only one type of electroporator and a single protocol would be ideal. However, the development of a standardized ECT protocol for npSCCs in cats was not included among the aims of the present study and we believe that the variability of instrumentations and protocols reflects the reality of clinical practice. Nevertheless, it would be interesting to conduct a prospective study including a comparison of different electroporators and protocols used in order to establish an ideal ECT procedure that would guarantee the best outcome for the patient. Moreover, although the local toxicity scoring system has been applied effectively in previous studies, its subjective nature is another limitation of this study.

## 5. Conclusions

The results of our study confirm that ECT is an effective and safe treatment for feline npSCCs and it could thus be considered one of the treatments of choice, especially in low-stage tumors. In our study population, the statistical analysis identified that tumor size and local treatment response were the variables that most influenced survival time and tumor recurrence or progression. This thus confirms that early diagnosis is crucial for these locally invasive carcinomas located on the nasal planum, and that the results of the first treatment are crucial for a successful patient outcome.

## Figures and Tables

**Table 1:** Facility enrolled, electroporator type and its electrical characteristics, type of electrode and anesthesia protocol adopted.

Facility (n° Cats)	Electroporator	ECT Frequency (Hz) (N° Cats)	Amplitude to Electrode Distance Ratio (V/cm) (N° Cats)	Electrode (N° Cats)	Anesthesia Protocol
SVG ^1^ (12)	Electrovet EZ ^7^	500 (12)	1000 (10)	N ^11^ (12)	Premedication: Dexmedetomidine 2 mcg/kg, IM ^15^ and Methadone 0.2 mg/kg, IM Induction: Propofol 2 mg/kg, IV Maintenance: Isoflurane
			1300 (2)		
AVC ^2^ (10)	Oncovet ^8^	1 (8)	1200 (10)	N ^12^(10)	Premedication/induction: Medetomidine 0.10–0.15 mg/kg, IM and Butorphanol 0.1–0.5 mg/kg, IV; Atipamezole 0.5 mL/kg, IV to reverse
		5000 (2)			
VCB ^3^ (26)	VET CP 125 ^9^	5000 (6)	1000 (6)	N ^13^ (26)	Premedication: Methadone 0.3 mg/kg, IM Induction: Propofol 5 mg/kg, IV Maintenance: Isoflurane.
	BTX ECM 830 ^9^	10 (20)	1300 (20)		
OVG ^4^ (10)	Electrovet EZ ^7^	500 (10)	1200 (10)	N ^11^ (9)	Premedication: Methadone 0.2mg/kg, IM or Butorphanol 0.2 mg/kg, IM both associated with Ketamine 5 mg/kg, IM and Dexmedetomidine 40 mcg/kg, IM (Atipamezole to reverse) Induction: Propofol 4.5mg/kg, IV Maintenance: Isoflurane
				P ^14^ (1)	
CVM ^5^ (2)	Electrovet S13 ^7^	1 (1)	1300 (2)	P ^14^ (2)	Premedication: Medetomidine 0.001 mg/kg, IM, Midazolam 0.2 mg/kg, IM and Methadone 0.2 mg/kg, IM Induction: Propofol 4.5 mg/kg, IV Maintenance: Isoflurane
GVS p ^6^ (1)	Cliniporator ^10^	5000 (1)	400 (1)	N ^12^(1)	Premedication: Dexmedetomidine 0.04 mg/kg, IM (Atipamezole to reverse) Induction: Propofol 4 mg/kg, IV Maintenance: Isoflurane

^1^ Veterinary Facility Dr. Enrico Sponza, Genova, Italy; ^2^ Ashleigh Vet Clinic, Knaresborough, UK; ^3^ Vet Câncer, Sao Paolo, Brasil; ^4^ Oncovet Group, Rome, Italy; ^5^ Centro Veterinario Meranese, Bolzano, Italy; ^6^ Veterinary Oncology Services, PLLC at Guardian Veterinary Specialists, New York, USA; ^7^ LEROY Biotech, St-Orens-de-Gameville, France; ^8^ Cyto Pulse Sciences, Holliston, USA; ^9^ Vet Câncer/IMPLASTIC, São Paulo, Brazil; ^10^ IGEA, Carpi, Italy; ^11^ Two parallel rows with 4 needles each, 5.9-mm-apart from each other and 15 mm in length; ^12^ Two parallel rows with 6 needles each, 6-mm-apart from each other and 10–25 mm in length; ^13^ Two parallel rows with 3 needles each, 3-mm-apart from each other and 25 mm in length; ^14^ Plate electrode, L-shaped 15 × 10 mm, each 2 mm in diameter and with 8-mm distance between the two electrodes; IM—Intramuscular; IV—Intravenously.

**Table 2:** Facility enrolled, tumor size range and, for each cat, the number of ECT treatments received, the local treatment response and the toxicity along with the post-ECT therapy.

Facility/Electroporator (N° Cats)	Size Range (cm)	N° of ECT (N° Cats)	Outcome (N° Cats)	Toxicity (N° Cats)	Post-ECT Therapy
SVG/Electrovet EZ (12)	0.2–4	1 ECT (4) 2 ECT (6) 3 ECT (1) 4 ECT (1)	CR ^1^ (7) PR ^2^ (5)	0 (11) 3 (1)	If necessary: Meloxicam single dose 0.2 mg/kg, then for a few days, as needed, 0.1 mg/kg, Amoxicillin and clavulanic acid 20 mg/kg.
AVC/Oncovet (10)	0.3–3	1 ECT (10)	CR ^1^ (9) PR ^2^ (1)	0 (1) 2 (7) 3 (1) 4 (1)	Amoxicillin and clavulanic acid 7–10 mg/kg, IV one dose, then Clindamycin 5.5 mg/kg, PO for 7 days, Meloxicam 0.3 mg/kg, SC, Dexamethasone 0.3 mg/kg, SC single dose 24 h after ECT.
VCB/VET CP 125 (6)	2.1–5	1 ECT (4) 2 ECT (1) 3 ECT (1)	CR ^1^ (1) PR ^2^ (5)	4 (3) 5 (4)	After ECT: Dipyrone 25 mg/kg, PO and Ketoprofen 1 mg/kg, PO (single dose); Home: Tramadol 1 mg/kg, PO SID 3 days, Dipyrone 25 mg/kg, PO SID 3–5 days, Ketoprofen 1 mg/kg, PO SID 3–5 days and Amoxicillin and clavulanic acid 15 mg/kg, PO BID 7 days. Persisting necrosis (toxicity grade 4 or 5): Amoxicillin and clavulanic acid 15 mg/kg, PO BID 7 days. Pain: Tramadol 1 mg/kg, PO SID 3 days (severe cases), Dipyrone 25 mg/kg, PO SID 3-5 days, Ketoprofen 1 mg/kg, PO SID 3–5 days. Severe cases: hospitalization 1-3 days with Methadone 0.1 mg/kg, SC BID, Dipyrone 25 mg/kg, IV SID, Ketoprofen 1 mg/kg, SC SID 3–5 days, Ampicillin and sulbactam 15 mg/kg BID 7 days IV. If hospitalization less than 7 days, antibiotic is replaced by Amoxicillin and clavulanic acid 15 mg/kg PO BID until the protocol is completed.
VCB/BTX ECM 830 (20)	0.8–3.6	1 ECT (12) 2 ECT (5) 3 ECT (3)	CR ^1^ (14) PR ^2^ (6)	2 (2) 3 (1) 4 (10) 5 (7)	
OVG/Electrovet EZ (10)	0.5–6	1 ECT (8) 2 ECT (1) 3 ECT (1)	CR ^1^ (6) PR ^2^ (2) SD ^3^ (2)	1 (2) 2 (5) 3 (1) 4 (1) 5 (1)	Meloxicam 0.05 mg/kg, PO and Amoxicillin and clavulanic acid 20 mg/kg, PO for 5–7 days. For wound cleaning and crust removal: saline and local ointments: VEA cream PF^®^ (antioxidant cream with Vit. E and Polyphenol), One Vet spray^®^ (neem oil, St John’s wort oil and olive oil), Iruxol^®^ (collagenase and chloramphenicol) or Hypermix^®^ (neem oil and St John’s wort oil)
CVM/Electrovet S13 (2)	0.6–3	1 ECT (1) 2 ECT (1)	CR ^1^ (2)	0 (1) 2 (1)	Meloxicam 0.3 mg/kg SC for 2–3 days.
GVS/Cliniporator (1)	0.5	2 ECT (1)	CR ^1^ (1)	0 (1)	None

^1^ No sign of the primary tumor [43]; ^2^ At least 30% reduction in the tumor but not the disappearance of the mass [43]; ^3^ Size of tumor showed no reduction or enlargement [43]; 0, no toxicity; 1, mild swelling; 2, swelling/necrosis <1 cm; 3, severe swelling; 4, deep necrosis; 5, severe swelling and tissue loss; SID—once a day; BID—twice a day; TID—three times a day; IM—Intramuscular; IV—Intravenously; PO—Per Os; SC—Subcutaneous. For the veterinary facilities involved in the study, refer to the legend in Table 1.

**Table 3:** Univariate and multivariate analyses for the relationship between variables and tumor-specific survival.

UNIVARIATE—Logistic Binary Regression					
	***Tumor-Specific Survival (N° Cats)***				
	**Yes**	**No**			
**Variables**			**^1^ p**	**^2^ OR**	**^3^ 95% CI**
Gender					
^4^ MN	9	21	0.187	2.04	0.71–5.9
^5^ FN	14	16	Reference category		
Local treatment response					
^6^ CR	4	35	Reference category		
^7^ PR	17	2	<0.001	74.4	12.37–447.05
^8^ SD	2	0	0.999	^9^ H	
Pulse frequency (Hz)					
1	1	9	0.035	0.07	0.01–0.82
10	5	15	0.072	0.2	0.04–1.15
500	12	10	0.70	0.72	0.14–3.78
5000	5	3	Reference category		
Amplitude to electrode distance ratio (V/cm)					
1000	11	5	Reference category		
1200	5	15	0.011	0.15	0.04–0.66
1300	7	17	0.016	0.19	0.05–0.74
Toxicity score					
0–2	10	20	Reference category		
3–5	13	17	0.43	1.53	0.45–4.36
Recurrence/progression					
Yes	13	6	0.002	6.72	2.02–22.34
No	10	31	Reference category		
Age (years)			0.806	1.02	0.85–1.23
Tumor size (cm)			0.074	1.52	0.96–2.40
**MULTIVARIATE—Logistic Binary Regression**					
**Variables**			**^1^ p**	**^2^ OR**	**^3^ 95%CI**
Local treatment response					
^6^ CR	4	35	0.001	Reference category	
^7^ PR	17	2	<0.001	179.28	10.97–2938.89
^8^ SD	2	0	0.999	^10^ VH	
Recurrence/progression					
Yes	13	6	0.017	27.4	1.80–386.95
No	10	31	Reference category		

^1^*p* value; ^2^ Odds ratio; ^3^ 95% Confidence Interval; ^4^ Male neutered; ^5^ Female neutered; ^6^ Complete response; ^7^ Partial response; ^8^ Stable disease; ^9^ High (the 95% CI is not reported); ^10^ Very high (the 95% CI is not reported).

**Table 4:** Univariate and multivariate analyses for the relationship between variables and local treatment response.

UNIVARIATE—Logistic Binary Regression					
	**Local Treatment Response (N° Cats)**				
	**^6^ CR**	**^7^ PR/^8^ SD**			
**Variables**			**^1^ p**	**^2^ OR**	**^3^ 95% CI**
Gender					
^4^ MN	21	9	0.418	1.56	0.53–4.53
^5^ FN	18	12	Reference category		
Pulse frequency (Hz)					
1	9	1	0.035	0.07	0.01–0.82
10	14	6	0.122	0.26	0.46–1.44
500	13	9	0.301	0.42	0.08–2.20
5000	3	5	Reference category		
Amplitude to electrode distance ratio (V/cm)					
1000	7	9	Reference category		
1200	15	5	0.061	0.26	0.06–1.07
1300	17	7	0.092	0.32	0.09–1.20
Toxicity score					
0–2	24	6	Reference category		
3–5	15	15	0.018	4.00	1.27–12.5
Age (years)			0.34	1.10	0.91–1.33
Tumor size (cm)			0.001	3.08	1.59–5.96
**MULTIVARIATE—Logistic binary regression**					
**Variables**			**^1^p**	**^2^OR**	**^3^95%CI**
Tumor size (cm)			0.008	4.50	1.48–13.67

^1^*p* value; ^2^ Odds ratio; ^3^ 95% Confidence Interval; ^4^ Male neutered; ^5^ Female neutered; ^6^ Complete response; ^7^ Partial response; ^8^ Stable disease.

**Table 5:** Univariate and multivariate analyses for the relationship between variables and toxicity.

UNIVARIATE—Logistic Binary Regression					
	**Toxicity (N° Cats)**				
	**0–2**	**3–5**			
**Variables**			**^1^ p**	**^2^ OR**	**^3^ 95% CI**
Gender					
^4^ MN	17	13	0.187	2.04	0.71–5.9
^5^ FN	13	17	Reference category		
Pulse frequency (Hz)					
1	8	2	0.29	0.08	0.01–0.77
10	2	18	0.32	3,00	0.34–26.19
500	18	4	0.008	0.07	0.01–0.51
5000	2	6	Reference category		
Amplitude to electrode distance ratio (V/cm)					
1000	10	6	Reference category		
1200	15	5	0.421	0.56	0.13–2.33
1300	5	19	0.01	6.30	1.54–26.0
Age (years)			0.077	0.84	0.70–1.02
Tumor size (cm)			<0.001	5.84	2.43–14.04
**MULTIVARIATE—Logistic Binary Regression**					
**Variables**			**^1^ p**	**^2^ OR**	**^3^ 95%CI**
Tumor size (cm)			0.005	3.75	1.49–9.44

^1^*p* value; ^2^ Odds ratio; ^3^ 95% Confidence Interval; ^4^ Male neutered; ^5^ Female neutered.

## Data Availability

Data available on request due to restrictions, e.g., privacy or ethical. The data presented in this study are available on request from the corresponding author. The data are not publicly available due to the multicentric nature of the study and the inclusion of various facilities from all over the world and to guarantee the privacy of the presented data.
